# Effects of respiratory and cardiac motion on estimating radiation dose to the left ventricle during radiotherapy for lung cancer

**DOI:** 10.1002/acm2.13855

**Published:** 2022-12-23

**Authors:** Alireza Omidi, Elisabeth Weiss, John S. Wilson, Mihaela Rosu‐Bubulac

**Affiliations:** ^1^ Department of Biomedical Engineering College of Engineering Virginia Commonwealth University Richmond Virginia USA; ^2^ Department of Radiation Oncology Virginia Commonwealth University Health System Richmond Virginia USA; ^3^ Pauley Heart Center Virginia Commonwealth University Health System Richmond Virginia USA

**Keywords:** cardiac motion, cardiotoxicity, image registration, radiotherapy, respiratory motion

## Abstract

**Purpose:**

Establish a workflow to evaluate radiotherapy (RT) dose variation induced by respiratory and cardiac motion on the left ventricle (LV) and left ventricular myocardium (LVM).

**Methods:**

Eight lung cancer patients underwent 4D‐CT, expiratory T1‐volumetric‐interpolated‐breath‐hold‐examination (VIBE), and cine MRI scans in expiration. Treatment plans were designed on the average intensity projection (AIP) datasets from 4D‐CTs. RT dose from AIP was transferred onto 4D‐CT respiratory phases. About 50% 4D‐CT dose was mapped onto T1‐VIBE (following registration) and from there onto average cine MRI datasets. Dose from average cine MRI was transferred onto all cardiac phases. Cumulative cardiac dose was estimated by transferring dose from each cardiac phase onto a reference cine phase following deformable image registration. The LV was contoured on each 4D‐CT breathing phase and was called clinical LV (cLV); this structure is blurred by cardiac motion. Additionally, LV, LVM, and an American Heart Association (AHA) model were contoured on all cardiac phases. Relative maximum/mean doses for contoured regions were calculated with respect to each patient's maximum/mean AIP dose.

**Results:**

During respiration, relative maximum and mean doses on the cLV ranged from −4.5% to 5.6% and −14.2% to 16.5%, respectively, with significant differences in relative mean doses between inspiration and expiration (*P* < 0.0145). During cardiac motion at expiration, relative maximum and mean doses on the LV ranged from 1.6% to 59.3%, 0.5% to 27.4%, respectively. Relative mean doses were significantly different between diastole and systole (*P* = 0.0157). No significant differences were noted between systolic, diastolic, or cumulative cardiac doses compared to the expiratory 4D‐CT (*P* > 0.14). Significant differences were observed in AHA segmental doses depending on tumour proximity compared to global LV doses on expiratory 4D‐CT (*P* < 0.0117).

**Conclusion:**

In this study, the LV dose was highest during expiration and diastole. Segmental evaluation suggested that future cardiotoxicity evaluations may benefit from regional assessments of dose that account for cardiopulmonary motion.

## 1 INTRODUCTION

Radiotherapy (RT) is a common and effective approach to treat patients with lung cancer using focal ionizing radiation. However, unwanted radiation to adjacent healthy tissues due to proximity to the tumour and current limitations in accounting for cardiopulmonary motion can lead to undesirable side effects. Unintended cardiovascular exposure is important not only for patients with lung cancer, but also other thoracic neoplasms (e.g., breast cancer, esophageal cancer), where depending on the target location, radiation beams that pass through the heart and great vessels can induce myocardial and endothelial damage and cause cardiovascular toxicity. For example, it has been shown for each 1 Gy increase in heart mean dose, the likelihood of major coronary events linearly increases by 7.4%.^1^ and for valves receiving doses > 40 Gy the rate of valvular heart disease increases by a factor 11.8.^2^

Major contributors to the degree of cardiovascular exposure during thoracic RT include interfraction and intrafraction motion. Interfraction motion (that is, changes in the position of tissues between radiation fractions) due to changes in patient's body habitus, tumour shrinkage/progression, and patient set‐up can be improved by using image‐guided RT (IGRT).^3^ In IGRT, different imaging techniques are used during the course of RT treatment to correct for interfraction motion by comparing the acquired image with the simulation image via image registration and making the proper adjustments (e.g., movement of treatment table) to improve the precision and accuracy of the delivery of radiation.^4^ A more challenging problem is the displacement of internal organs due to cardiopulmonary motion, also known as intrafraction motion,^4^ which requires more sophisticated approaches for quantification, such as cine imaging with high temporal resolution.^3^

Currently, the assessment of the respiratory motion and the subsequent radiation exposure of tumour and organs at risk is commonly performed by using 4D‐CT (which acquires multiple breathing phases throughout the respiratory cycle) instead of conventional 3D‐CT (which acquires a single ‘snapshot’ image that neglects motion). Notably, even though 4D‐CT captures motion due to the respiratory cycle, it does not assess changes due to cardiac motion, which is much faster than respiratory movement (60–100 beats‐per‐minute^5^ vs 12–18 breaths‐per‐minute^6^) and cannot be mitigated as with breathing management techniques. Both of these motions can impact the dose received by the tumour and the heart.

A few studies have investigated the effects of cardiac motion on the displacement of the heart and its substructures to improve RT treatment planning margins. In general, it is recommended to have a 5 mm margin to account for motion of all cardiac substructures during RT.^7^ For example, it has been shown that the maximum left ventricle (LV) displacement between systole and diastole measured via cardiac‐gated contrast‐enhanced CT images at breath‐hold was in the anterior‐to‐posterior direction (5 ± 1.5 mm).^8^ Cardiac motion also affects the accurate dose delivery of cardiac radioablation (CR). In one study, cardiac‐gated cine MR images at breath‐hold were used to measure the atrial fibrillation (AF) target motion due to cardiac contraction. Interestingly, AF CR target motion was noted to be prominent in the medial‐lateral direction (4–5 mm).^9^ however, this study did not report any dose measurements. Despite such reports on cardiac motion‐induced LV displacement, few studies have measured the dose received by the LV/left ventricle myocardium (LVM) at systolic and diastolic phases of the cardiac cycle. In one MR‐guided RT (MRgRT) study, the LVM dose was measured at systole and diastole using image registration between the planning CT and end‐diastolic/systolic cine MR images at breath‐hold among patients with various thoracic tumours. The mean LVM dose remained unchanged while the maximum dose increased at systole.^8^ In another assessment of LVM dose (with exclusion of the interventricular septum), breath‐hold cardiac‐gated 4D‐CT images were acquired for patients with esophageal carcinomas. The RT dose was registered from the planning 0% 4D‐CT to each cardiac phase to generate the dose distribution for all the phases. The LVM mean dose ranged between 6.7 ± 3.7 and 11.5 ± 3.8 Gy, but the authors did not report the actual cardiac phase where the maximum and minimum doses occurred.^9^ Finally, the LV dose variation was measured among left‐sided breast cancer patients using cardiac‐gated dual source CT (in deep inspiration breath‐hold). Higher LV maximum dose was reported at diastole vs. systole (26.7 Gy vs 21.5 Gy, *P* = 0.005).^10^ Nonetheless, the reported absolute doses depended on the prescription dose.

From these studies, it is evident that cardiac motion can impact the dose distribution on the LV and/or LVM. Though not measured in each study, the LVM dose variations might be more important than the LV alone as it quantifies the actual radiation delivered to the cardiac muscle, which can lead to myocardial injury.^10^, ^11^ More importantly, LVM segmentation has the potential to allow for regional dose calculations (e.g., using the 16‐segment American Heart Association (AHA) model) to divide the LVM into regional subsegments at basal, mid‐cavity, and apical short‐axis slices,^12^ which has not been included in prior studies. Though many prior studies have utilized CT to assess the LV/LVM dose distribution, short‐axis cine magnetic resonance (MR) images can provide excellent tissue contrast (compared to non‐contrast CT)^13^ between the LV wall and the blood pool, enabling a potentially more accurate estimation of radiation dose to the cardiac muscle. However, a challenge in utilizing the benefits of cine MR has been that RT planning has traditionally been performed on 4D‐CT, not MRI, which requires image registration algorithms to transform the dose between imaging modalities. Despite the importance of both cardiac and respiratory motion on the dose delivered to the LV/LVM, to the best of our knowledge, no prior studies have compared the effects of respiratory and cardiac motion on LV dose variations. Notably, lack of cardiac‐motion gating in the 4D‐CT dataset would result in capturing an average volume of the structures affected by cardiac motion (e.g., LV). To distinguish the differences between the LV volumes from cardiac‐gated and non‐cardiac‐gated sequences, the LV from non‐cardiac‐gated 4D‐CT datasets will be denoted as clinical LV (cLV) herein, while the volumes from cardiac‐gated MRI sequences will be denoted as LV/LVM.

Therefore, the primary aim of this study was to implement an image registration‐based workflow to comparatively explore the following aims: (1) the variations in radiation dose to the cLV across the respiratory cycle, (2) the variations in radiation dose to the LV/LVM across the cardiac cycle at expiratory breath‐hold, (3) a novel approach to quantify regional and global cumulative dose to the LV/LVM via deformable image registration (DIR) that accounts for patient‐specific cardiac motion.

## 2 METHODS AND MATERIALS

### 2.1 Patients

This study was approved by the local institutional review board (IRB) and each patient provided written informed consent prior to enrollment. Eight patients with lung cancer who were scheduled to receive at least 5 Gy of radiation to ≥10% of the volume of the heart were recruited for this prospective observational study. Patients were between 58 and 77 years old and had a mean prescribed dose of 62 (50–66) Gy at 2 Gy per fraction. Table 1 shows individual patient characteristics.

### 2.2 Treatment planning

Each patient underwent a non‐contrast free‐breathing 4D‐CT scan using a Brilliance Big Bore CT (Philips Healthcare, Cleveland, OH) as part of treatment planning to account for the respiratory motion during radiation delivery. Key parameters included: slice thickness 3 mm, pixel size 1.17 × 1.17 mm, kilovoltage peak (kVp) 120 kV, exposure time 6347 ms, X‐ray tube current 95 mA, helical acquisition. The respiratory motion was detected with a Philips bellows device. Ten breathing phases were reconstructed (0%–90%) from the acquired dataset with 0% and 50% labelled as end‐inspiration and end‐expiration, respectively. The pixel intensities across all breathing phases of the 4D‐CT dataset were averaged to form an average intensity projection (AIP) image. All treatment plans were designed based on the AIP.^14^

The gross tumour volume (GTV) was contoured on the 30% phase of the 4D‐CT and propagated over all phases to create an iGTV (internal gross tumour volume). The internal target volume (ITV) was generated by expanding the iGTV by 6–8 mm depending on histopathology and by excluding uninvolved structures such as large vessels, esophagus, heart, bone or chest wall. The ITV was then expanded 5 mm for the planning target volume (PTV).^15^ Normal tissues (lungs, heart, esophagus, spinal cord, brachial plexus) were contoured on the AIP image. Plans were optimized to achieve PTV V100% prescription dose ≥ 95%. Normal tissue dose constraints included the following parameters: spinal cord maximum dose ≤ 45 Gy, brachial plexus maximum dose ≤ 66 Gy, mean lung dose ≤ 20 Gy, lungs V_20Gy_ (volume of the lung receiving ≥ 20 Gy) ≤ 30% of the prescribed dose, lungs V_30Gy_ (volume of the lung receiving ≥ 30 Gy) ≤ 20%, mean esophagus dose ≤ 34 Gy, esophagus V_60Gy_ ≤ 17%, esophagus maximum dose ≤ 105% of prescription dose, heart V_45Gy_ ≤ 30%, heart V_30Gy_ ≤ 40%, mean heart dose ≤ 20 Gy. Volumetric modulated arc therapy (VMAT) technique was used to deliver the prescribed dose to PTV using daily treatments Monday through Friday over 5 to 6.5 weeks. Treatment planning was performed using ECLIPSE (version 16.01.04, Varian Medical Systems, Palo Alto, CA, USA) with Acuros XB dose calculation algorithm (Acuros External Beam, Version 15.6.06).

### 2.3 MR imaging

#### 2.3.1 Cine MRI

Cardiac‐gated gradient echo (GRE) cine MR images were acquired on a 3T MRI platform (MAGNETOM Vida from Siemens Healthineers) at expiratory breath‐hold to capture 25 phases over the cardiac cycle at 8–10 short‐axis slices of the LV from apex to the root of the aortic valve. Relevant MR parameters include: slice thickness 1 cm, time to echo (TE) 1.24 ms, repetition time (TR) 42.9 ms, flip angle 40°, echo train length (ETL) 1, pixel size 1.33 × 1.33 mm.

#### 2.3.2 T1‐ volumetric interpolated breath‐hold examination (VIBE) MRI

Following cine MR acquisition, axial 3D T1‐VIBE MRI was acquired for the whole chest during an expiratory breath‐hold. Key T1‐VIBE imaging parameters include: slice thickness 2 mm, TE 1.33 ms, TR 4.1 ms, ETL 2, flip angle: 9°, pixel size 1.19 × 1.19 mm. All MR images were acquired in breath‐hold to reduce motion artifacts.

#### 2.3.3 Contouring

The cLV was manually contoured on each respiratory phase of 4D‐CT (0%–90%) based on published guidelines^16^ and anatomical landmarks from the level of the mitral and aortic valves to the apex, including the septum and free wall. In cine MR images, all short‐axis slices associated with individual cardiac phases were grouped together to form a 3D volumetric LV structure at each of the 25 cardiac phases. Following the same anatomical landmarks, the LV and LVM (LV without blood pool) were manually contoured on all cardiac phases (1–25) of the cine MR images (including systole, diastole, and the reference cine MR phase (i.e., phase 1)); each LVM contour was further segmented according to the 16‐segment AHA model.^12^ Systolic and diastolic phases were chosen per Society of Cardiovascular Magnetic Resonance (SCMR) task report as the smallest and largest global LV blood volume pools, respectively,^17^ with visual monitoring of the mid‐ventricular slice blood pool when it reaches its minimum and maximum.^18^ Note that the 4D‐CT dataset could not be divided into the 16 segments of the AHA model due to insufficient contrast between the intraventricular blood pool and the LV wall and the lack of local LV short‐axis views in the CT data (which was acquired as anatomical axial slices). All segmentations were performed on MIM software (MIM Software Inc., Cleveland, OH, USA).

#### 2.3.4 Image registration and dose transfer

Image registration is a process that utilizes an algorithm to map the data from one coordinate space onto another coordinate space.

For this study, MIM software was used to perform the required registrations. The DIR algorithm in MIM is an intensity‐based free‐form algorithm, which consists of a coarse‐to‐fine multi‐resolution approach that searches for point‐by‐point corresponding locations using a grid of control points on the primary dataset. Initially, a coarse grid is used to account for gross differences, followed by higher resolution comparisons to address local changes over smaller scales. Finally, a gradient descent‐based approach is used as an optimization algorithm, and the degree of match is assessed by an intensity‐based sum of square differences strategy.^19^, ^20^, ^21^ The accuracy of registration of 4D‐CT and T1‐VIBE MR images in MIM software (i.e., compliance with American Association of Physicists in Medicine (AAPM) task group 132 report requiring mean distance to agreement (MDA) < 3 mm and Dice > 0.8^22^) was demonstrated in a previous study (MDA < 2.6 mm and Dice > 0.85).^21^

The purpose of image registration in this study was (1) to facilitate the estimation of dose at various respiratory and cardiac phases, and (2) to evaluate the cumulative dose over the cardiac cycle. More details are provided below.

(1) The 4D‐CT images and the derived AIP set (used for treatment planning) are all in the same reference system, thus, the dose on each phase was estimated by ‘transferring’ the planned dose from the AIP onto 4D‐CT series, under the assumption that the dose is mostly unperturbed by the change in the anatomy; the concept of transfer is supported by the fact that the individual respiratory phases are inherently rigidly registered. In addition, it has been shown that the dosimetry map calculated on each respiratory phase is equivalent to the AIP dose, except for high iso‐dose lines adjacent to the tumour due to the build‐up effect and rapid dose fall‐off at the interface between tumour and lung.^23^, ^24^ The same line of reasoning applies to estimation of the dose on each cardiac phase, using the dose from the average cine MRI as input. However, it should be noted that transfer of the dose from AIP onto average cine MRI is not directly possible given that the cine MR images have a limited field of view (FOV), which makes it impossible to establish manually or automatically a proper frame of reference correspondence between the 4D‐CT and cine MRI. This issue was addressed by using a bridging image set, the T1‐VIBE MRI, which has the same frame of reference as the cine MRI (thus the FOV limitation of the cine MRI is overcome) and covers the entire region of interest (which allows for the multi‐modality registration with the 4D‐CT series, rigidly or non‐rigidly, depending on what was deemed necessary on a patient‐by‐patient basis).
(2) The cumulative doses over the cardiac cycle were accomplished by transfer of the dose to a reference cardiac phase; this transfer was always performed using deformable registration.

#### 2.3.5 Respiratory motion effect on the cLV

**FIGURE 1:**
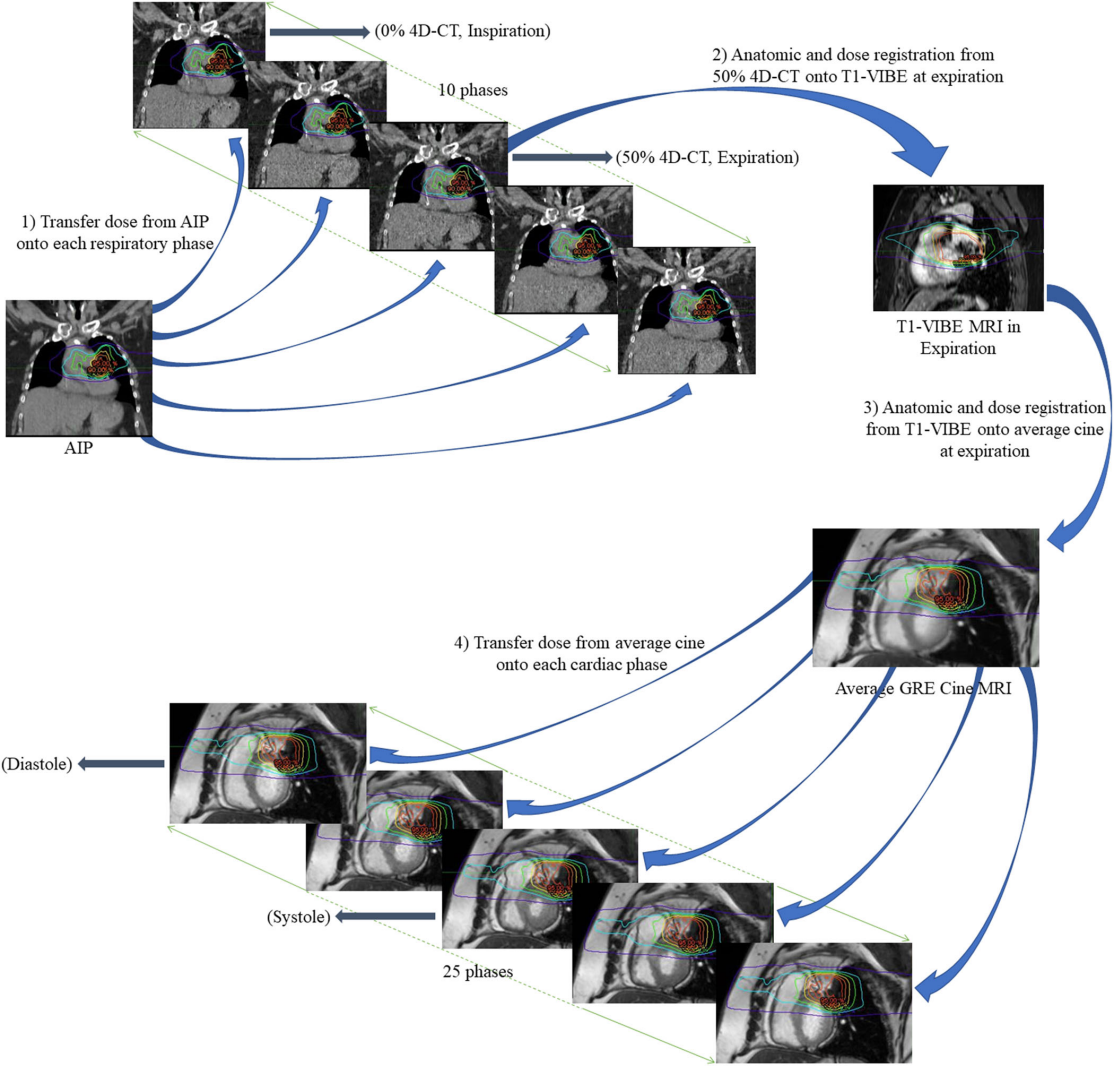
(1) Workflow for analyzing the respiratory effect in which dose from the AIP image set is transferred onto each respiratory phase of the 4D‐CT dataset (0%‐90%). (2) Dose transfer from 50% 4D‐CT onto T1‐VIBE at expiration. (3) Dose transfer from T1‐VIBE onto average cine MRI, (4) workflow for analyzing the cardiac effect in which dose from average cine MRI is transferred onto each cardiac phase (1‐25)

The dosimetry map planned on the AIP dataset was transferred onto each respiratory phase of the 4D‐CT (0%–90%), since there is no change in the position of the body as a whole during the 4D‐CT acquisition. The maximum and mean doses were determined for the contoured cLV volume using a dose volume histogram (DVH). The schematic of the workflow is shown in Figure 1.

In addition, the largest cLV displacements in superior‐to‐inferior (SI), right‐to‐left (RL), and anterior‐to‐posterior (AP) directions during respiratory motion were measured using the displacement of the centroid of the cLV between inspiratory and expiratory phases. The 3D magnitude of displacement was calculated as the square‐root of the sum of the squares of each directional displacement.

#### 2.3.6 Cardiac motion effect on the LVM and LV at expiration

In order to quantify the variation in dose due to cardiac motion, the dose from the 50% 4D‐CT (expiration) was mapped onto the expiratory volumetric T1‐VIBE following image registration (rigid or deformable based on degree of deformation observed in each patient) and from there onto the average cine MRI following a rigid registration between T1‐VIBE and average cine MRI (Figure 1). Finally, the dose on the average expiratory cine MRI was transferred onto all 25 cardiac phases, including systole and diastole, since these phases share the same body position relative to the radiotherapy (Figure 1). The maximum and mean doses were obtained for the contoured LV and LVM using a DVH.

Furthermore, the largest LV displacement in the SI, RL, AP directions during cardiac motion was measured by calculating the displacement of the centroid of the LV between systolic and diastolic phases. The magnitude of displacement was measured as described above.

#### 2.3.7 Cumulative LVM and LV dose during the cardiac cycle on reference cine MR at expiration

**FIGURE 2:**
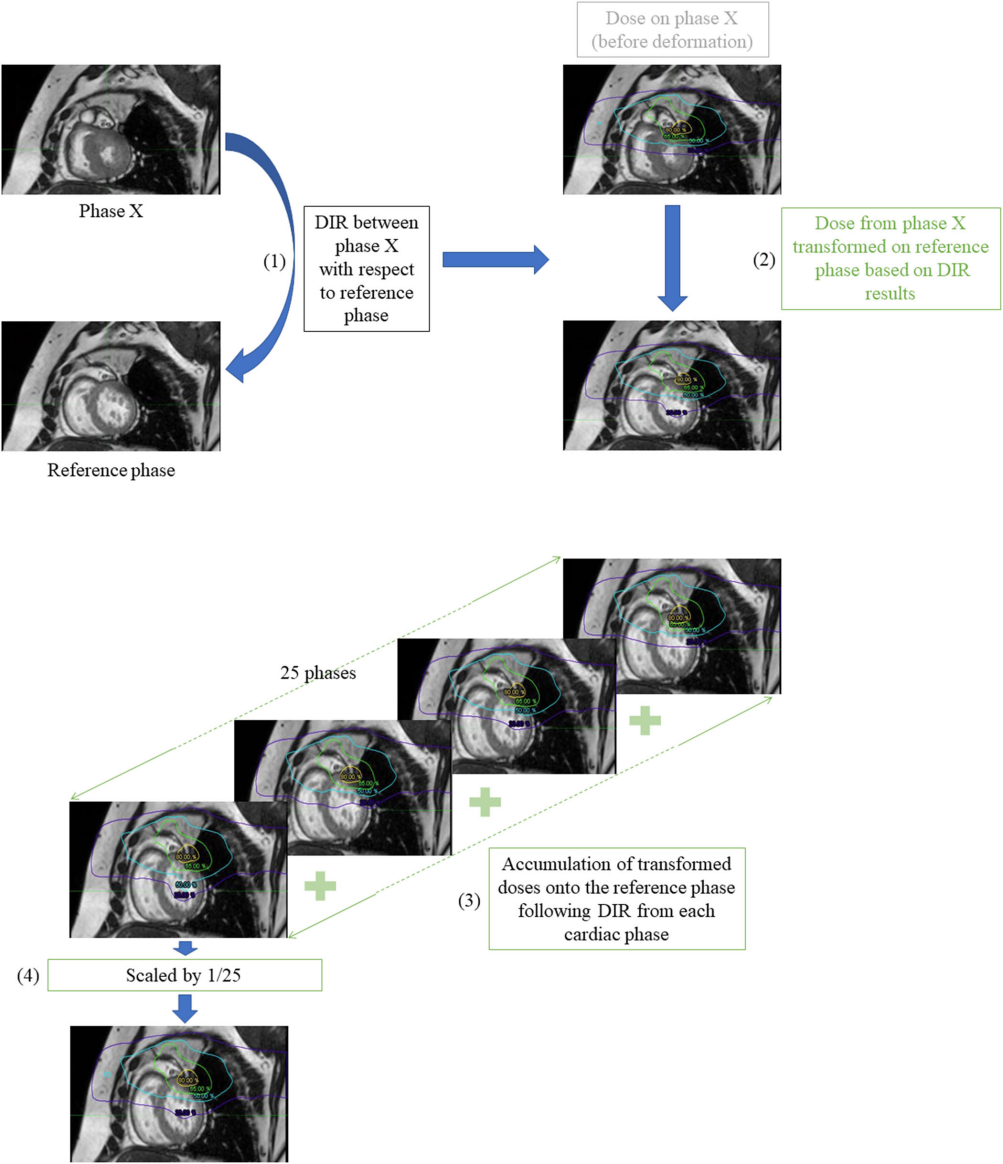
(1), (2) Workflow for estimation of cumulative dose using dose transformations from each cardiac phase (all 25 cardiac phases) onto the reference cine phase following a DIR of the anatomy between each phase and the reference phase. (3), (4) Accumulation of the transformed doses on the reference cine phase from all 25 cardiac phases and scaling by 1/25 to estimate total cumulative dose over the cardiac cycle

In order to estimate the cumulative global and regional dose across all cardiac phases from the expiratory cine MRI, each cardiac phase (and respective dosimetry map) underwent a DIR process with respect to the reference cine MRI (i.e., phase 1). Having mapped all doses from each phase to the reference anatomy, doses from all cardiac phases were equally weighted in the cumulative dose (scaled by the number of phases (25)), because all the cardiac phases required the same amount of time to be collected. The global and regional maximum and mean doses of the LV and LVM were derived from the DVH. This workflow is depicted in Figure 2. All transformation/registration processing of the doses was performed using MIM software.

Throughout this study, the adequacy of image registration was assessed by visual inspection of alignments and matching between two datasets on a patient‐specific basis, which is regarded as the ultimate reliable tool in hand.^25^ Manual adjustments (e.g., rotation/translation) were applied in cases of initial misalignment. The ‘Reg Refine’ tool in MIM software enables assessment and improvement of image registration accuracy.^19^

#### 2.3.8 Data analysis and statistics

Comparisons of estimated dose variations were performed globally for the cLV/LV data, both globally and/or regionally for the LVM data. For global analyses, the relative maximum and mean doses were compared between (1) the respiratory phases on the cLV, (2) the cardiac phases including systole and diastole on the LVM/LV at expiration, (3) systolic and diastolic cardiac phases at expiration on the LV and the corresponding breathing phase (50% 4DCT) on the cLV, and (4) the cumulative LVM/LV dose on the cardiac‐gated expiratory reference cine MR and the cLV dose on the non‐cardiac‐gated expiratory 4D‐CT (50%) dataset. In addition, the range of cLV/LV maximum and mean dose variation over the respiratory cycle and cardiac cycle were determined to assess the magnitude of cLV/LV dose differences between the respiratory phases with intrinsic cardiac motion and cardiac phases at breath‐hold. For regional analysis, the cumulative dose on the reference cine MR phase for each region of the AHA model was compared to the single mean dose value from the non‐cardiac gated expiratory 4D‐CT (50%) dataset. To better compare the doses between various modalities, patients, and processing techniques, all dose values were reported as doses relative to the AIP dose measured on the cLV (except for the cardiac motion effect, where doses were normalized to both AIP and 50% 4D‐CT to capture the cardiac motion effect alone) using Equations (1) and (2). All reported dose values are for the population average unless specified.

(1) $$\begin{array}{ccc} & & \;\\ & \mathrm{Rela}\mathrm{tive}\;\mathrm{maxi}\mathrm{mum}\;\mathrm{dose} & =\frac{\left(\mathrm{maxi}\mathrm{mum}\;\mathrm{dose}-\mathrm{maxi}\mathrm{mum}\;\mathrm{AIP}\;\mathrm{dose}\right)}{\mathrm{maxi}\mathrm{mum}\;\mathrm{AIP}\;\mathrm{dose}}\;\;*100 \end{array}$$

(2) $$\begin{array}{ccc} & & \;\\ \mathrm{Rela}\mathrm{tive}\;\mathrm{mean}\;\mathrm{dose} & = & \frac{\left(\mathrm{mean}\;\mathrm{dose}-\mathrm{mean}\;\mathrm{AIP}\;\mathrm{dose}\right)}{\mathrm{mean}\;\mathrm{AIP}\;\mathrm{dose}}\;\;*100 \end{array}$$

ANOVA (unequal variance) was used to explore significant differences in the global relative maximum dose or relative mean dose among respiratory phases. Student's *t*‐test was used to compare differences in relative maximum dose or relative mean dose between (1) systole versus diastole on expiratory cine MR, (2) systole versus 50% 4D CT, (3) diastole versus 50% 4DCT, and (4) regional/global cumulative dose on expiratory cine MR versus expiratory non‐cardiac‐gated 4D‐CT (50%). Student's *t*‐test was also used to compare the respiratory‐motion‐induced cLV displacement and cardiac‐motion‐induced LV displacement. All the analyses were done using JMP software (version Pro 14, SAS Institute Inc., NC, USA) with significance level of *P* ≤ 0.05.

## 3 RESULTS

### 3.1 Respiratory motion effect on the cLV

**FIGURE 3:**
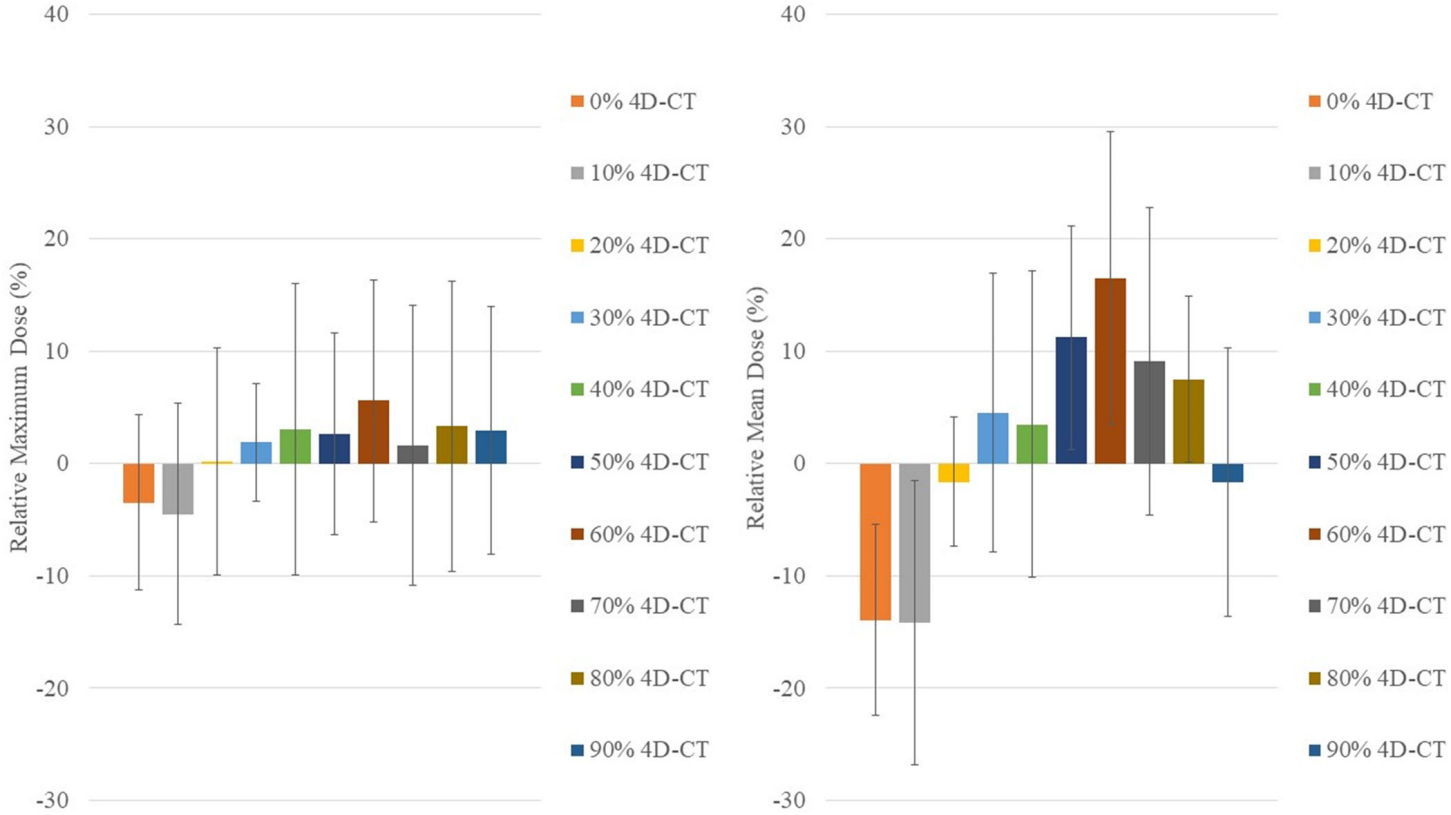
Relative maximum and mean dose values (± SD) on the cLV over the respiratory cycle on 4D‐CT, averaged over the patient population. AIP relative maximum and mean doses are zero

Table 2 shows the relative maximum and mean radiation dose (and range over the population for each value) on the cLV for each respiratory phase in the 4D‐CT, in addition to absolute dose value changes with respect to absolute AIP dose for each respiratory phase. Note that all doses are relative to the AIP for that patient; hence, the relative AIP dose is zero. The population‐averaged absolute maximum dose, absolute mean dose, relative maximum dose, and relative mean dose among the various breathing phases ranged between 35% and 37.8 Gy, 6.9% and 9.6 Gy, −4.5% and 5.6%, −14.2% and 16.5%, respectively. The cLV mean dose was minimal during inspiration (phases 0%–10%) and maximal during expiration (phases 50%–60%). Bar charts illustrating the variation of relative maximum and mean dose to the cLV over different respiratory phases are shown in Figure 3. Table 3 summarizes ANOVA tests for the relative mean dose among breathing phases and AIP. Significant differences were noted between 0% 4D‐CT and 50%‐60%‐70%‐80% 4D‐CT (*P* < 0.0145) and between 10% 4D‐CT and 50%‐60%‐70%‐80% 4D‐CT (*P* < 0.0125). No significant differences were observed for the relative maximum dose values among breathing phases (*P* > 0.78).

**TABLE 1:** Individual patient characteristics

Patient	Gender	Age	Prescribed dose (Gy)	Cancer stage	Tumour location
**1**	Female	67	64	III	Left lung (upper lobe), posterior to the heart
**2**	Male	62	66	III	Right lung (mid lobe), posterior to the heart
**3**	Female	59	60	III	Right lung (upper lobe), superior to the heart
**4**	Female	70	66	III	Left lung (upper lobe), superior to the heart
**5**	Male	62	50	IV	Left lung (upper lobe), superior to the heart
**6**	Male	62	66	IV	Left lung (upper lobe), superior to the heart
**7**	Female	77	66	III	Right lung (upper lobe), superior to the heart
**8**	Female	58	66	III	Right lung (mid lobe), superior to the heart

**TABLE 2:** The absolute and relative maximum and mean cLV dose differences across the respiratory cycle

	Relative maximum dose difference (%)			Absolute maximum dose difference (Gy)			Relative mean dose difference (%)			Absolute mean dose difference (Gy)		
	Average	Range		Average	Range		Average	Range		Average	Range	
**AIP**	0	0	0	0	0	0	0	0	0	0	0	0
**0% 4D‐CT**	− 3.5	− 19.1	7.3	− 0.8	− 3.7	0.6	− 13.9	− 26.2	− 0.6	− 1.2	−3.7	− 0.1
**10% 4D‐CT**	− 4.5	− 21.9	10.7	−1.2	−3.3	0.7	−14.2	−40.3	−1.3	−1.3	−3.9	−0.1
**20% 4D‐CT**	0.2	−17.3	22.3	0.1	−0.5	1.9	−1.6	−8.4	11.2	−0.2	−0.5	1.9
**30% 4D‐CT**	1.9	−5.3	14.0	0.5	−0.2	2.3	4.5	−17.3	27.7	0.1	−2.6	2.7
**40% 4D‐CT**	3.9	−10.9	36.0	0.9	−2.1	3.1	3.5	−25.9	18.0	0.1	2.3	2.1
**50% 4D‐CT**	2.7	−4	25.9	0.8	1.8	3.2	11.2	−1.6	25.6	0.8	−0.4	3.3
**60% 4D‐CT**	5.6	−4.4	31.4	1.6	−0.2	3.3	16.5	−1.2	33.2	1.4	−0.3	3.7
**70% 4D‐CT**	1.6	−14.2	31.4	0.4	−0.6	3.1	9.1	−8.6	30.4	0.6	−1.5	3.2
**80% 4D‐CT**	3.3	−3.5	37.4	0.7	0.9	2.7	7.5	0	23.0	0.6	−1.5	3.2
**90% 4D‐CT**	2.9	−4.1	31.9	0.6	−1.1	2.8	−1.7	−29.2	14.9	−0.4	−3.9	0.6

*Note*: Values are reported over the average population. The inspiratory phase was designated as 0%, the expiratory phase was designated as 50%. The absolute dose differences are the absolute dose values subtracted from the AIP dose. Relative doses were obtained from equation (1) and (2). The range of the population average of maximum absolute dose over the respiratory cycle was 35–37.8 Gy (individual range over all respiratory phases: 1.8‐70.4 Gy). The range of the population average of mean absolute dose over the respiratory cycle was 6.9‐9.6 Gy (individual range over all respiratory phases: 0.6‐27.2 Gy).

**TABLE 3:** P‐values for comparisons of the relative mean dose for 4D‐CT breathing phases on the cLV

	0% 4DCT	10% 4D‐CT	20% 4D‐CT	30% 4D‐CT	40% 4D‐CT	50% 4D‐CT	60% 4D‐CT	70% 4D‐CT	80% 4D‐CT	90% 4D‐CT
**AIP**	0.36	0.33	1	0.99	0.99	0.67	0.15	0.88	0.96	1
**0% 4D‐CT**	–	1	0.55	0.065	0.1	**0.0017**	**0.0001**	**0.006**	**0.0145**	0.55
**10% 4D‐CT**	–	–	0.51	0.057	0.09	**0.0014**	**0.0001**	**0.0051**	**0.0125**	0.52
**20% 4D‐CT**	–	–	–	0.99	0.99	0.48	0.075	0.73	0.88	1
**30% 4D‐CT**	–	–	–	‐	1	0.98	0.59	0.99	1	0.99
**40% 4D‐CT**	–	–	–	‐	‐	0.95	0.47	0.99	0.99	0.99
**50% 4D‐CT**	–	–	–	‐	‐	–	0.99	1	0.99	0.57
**60% 4D‐CT**	–	–	–	‐	‐	–	‐	0.97	0.9	0.073
**70% 4D‐CT**	–	–	–	‐	‐	–	‐	‐	1	0.73
**80% 4D‐CT**	–	–	–	‐	‐	–	‐	‐	‐	0.88

The cLV displacements (± standard deviation (SD)) (over the population) during respiratory motion in the SI, RL, AP directions were 0.4(± 0.3) cm, 0.3(± 0.3) cm, 0.2(± 0.1) cm, respectively, yielding a magnitude of displacement of 0.6(± 0.2) cm.

### 3.2 Cardiac motion effect on the LVM/LV at expiration

**FIGURE 4:**
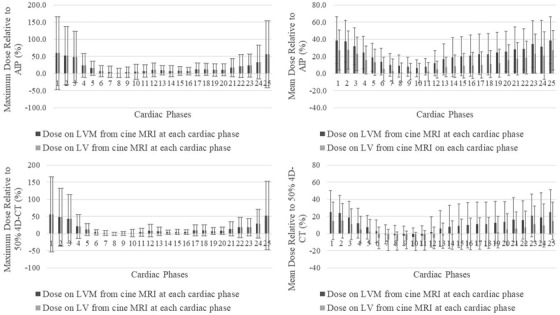
Maximum and mean LV and LVM dose values (± SD) on cine MRI relative to AIP (top row) and 50% 4D‐CT (bottom row) over the cardiac cycle at expiration breath‐hold, averaged over the patient population. Maximum LV/LVM dose ranged between 1.6–59.3% and ‐1.3–55.9% relative to AIP and 50% 4D‐CT, respectively. Mean LV dose ranged between 0.5–27.4%and ‐9.2–14.8% relative to AIP and 50% 4D‐CT, respectively.

**FIGURE 5:**
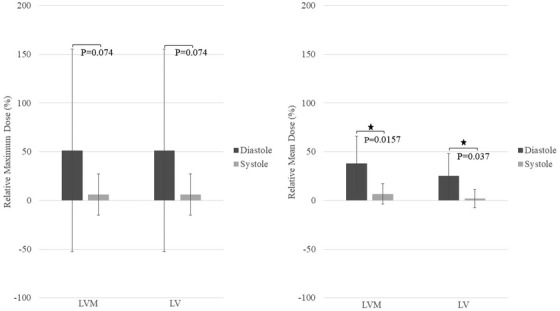
Relative maximum and mean dose values (± SD) on the LVM and LV at cardiac diastole and systole (over expiratory breath‐hold on cine MRI). Star signs demonstrate significant differences (P≤0.05)

Figure 4 shows the relative maximum and mean dose (± SD) for the LVM and LV throughout the whole cardiac cycle on expiratory breath‐hold cine MR relative to the original AIP and 50% 4D‐CT. For the LV, absolute maximum dose, absolute mean dose, maximum dose relative to AIP, and mean dose relative to AIP throughout the cardiac cycle ranged between 36.1 and 40.5 Gy, 8.5 and 10.5 Gy, 1.6% and 59.3%, and 0.5% and 27.4%, respectively. Absolute maximum dose and relative maximum dose were the same for LVM and LV, likely since the maximum dose was in the cardiac wall; however, the LVM absolute mean dose and relative mean dose varied between 9.2% and 11.4 Gy and 6% and 38.6%, respectively, due to the LVM excluding the blood pool. Relative to 50% 4D‐CT, maximum dose varied between −1.3% and 55.9% for both LV and LVM, mean dose ranged between −4.2% and 25% and −9.2% and 14.8% for LVM and LV, respectively (Figure 4). The LVM and LV mean dose were maximal at diastole (phases 1–2) and minimal at systole (phase 8–10). For both LVM and LV, significant relative mean dose differences were found between systole and diastole (LVM: diastole 37.8%, systole 6.8%, *P* = 0.0157; LV: diastole 25.1%, systole 2%, *P* = 0.037). No significant differences were noted in the relative maximum dose between diastole and systole for both LV and LVM (51.4% vs. 6% *P* = 0.074 for both). Figure 5 demonstrates the difference at diastole and systole for both LV and LVM.

Comparison of the LV relative maximum and mean dose variation throughout the cardiac cycle on cine MR at expiration versus the non‐cardiac gated expiratory 4D‐CT (50%) showed that even though there is variation in relative maximum and mean doses over all cardiac phases compared to the dose values on the corresponding breathing phase at 4D‐CT (50%), no significant differences were observed, including at systole and diastole (relative maximum dose at systole: 6% (cine MR) vs. 2.7% (50% 4D‐CT), *P* = 0.4; relative mean dose at systole: 2% vs. 11.2%, *P* = 0.14; relative maximum dose at diastole: 51.4% vs 2.7%, *P* = 0.24; relative mean dose at diastole: 25.1% vs. 11.2%, *P* = 0.2).

The LV displacement (±SD) (over the population) during cardiac motion was 0.4(± 0.4) cm, 0.2(± 0.1) cm, 0.4(± 0.4) cm in SI, RL, and AP directions, respectively, yielding a magnitude of displacement of 0.7(± 0.4) cm for the whole population.

### 3.3 Cumulative LVM dose over the whole cardiac cycle at expiration

### 3.4 Global analysis

**FIGURE 6:**
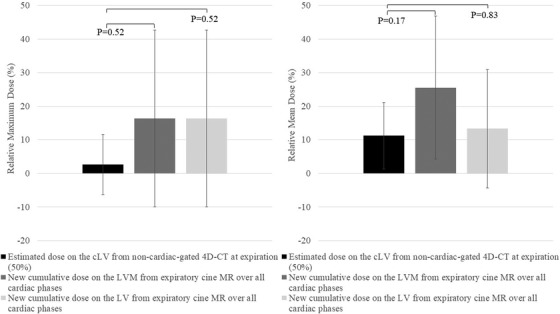
Relative maximum and mean dose values (± SD) of the new cumulative dose on the LVM and LV from expiratory cine MR over all cardiac phases versus the estimated dose on the cLV from non‐cardiac‐gated 4D‐CT at expiration (50%)

Figure 6 shows the differences in global relative maximum and mean cumulative doses estimated across all cardiac phases using expiratory cine MR (for the LV and LVM) compared to the corresponding non‐cardiac‐gated expiratory 4D‐CT (50%) (for the cLV) that neglects cardiac motion. Comparing the relative maximum cumulative dose of 16.4% for the LV or LVM to the relative maximum dose of 2.7% for the 50% 4D‐CT did not reach significance (*P* = 0.52). Comparison of the relative mean cumulative dose on the LV (13%) or the LVM (25.6%) to the relative mean dose on 50% 4D‐CT (11.2%) also did not reach significance (*P* = 0.83 and *P* = 0.17, respectively). In terms of absolute dose, maximum cumulative LV or LVM dose was 38.3 Gy versus the 37.0 Gy dose on cLV from 50% 4D‐CT. Mean cumulative LV and LVM doses were 9.5 Gy and 10.6 Gy, respectively compared to 9.2 Gy cLV dose on 50% 4D‐CT.

### 3.5 Regional analysis

**FIGURE 7:**
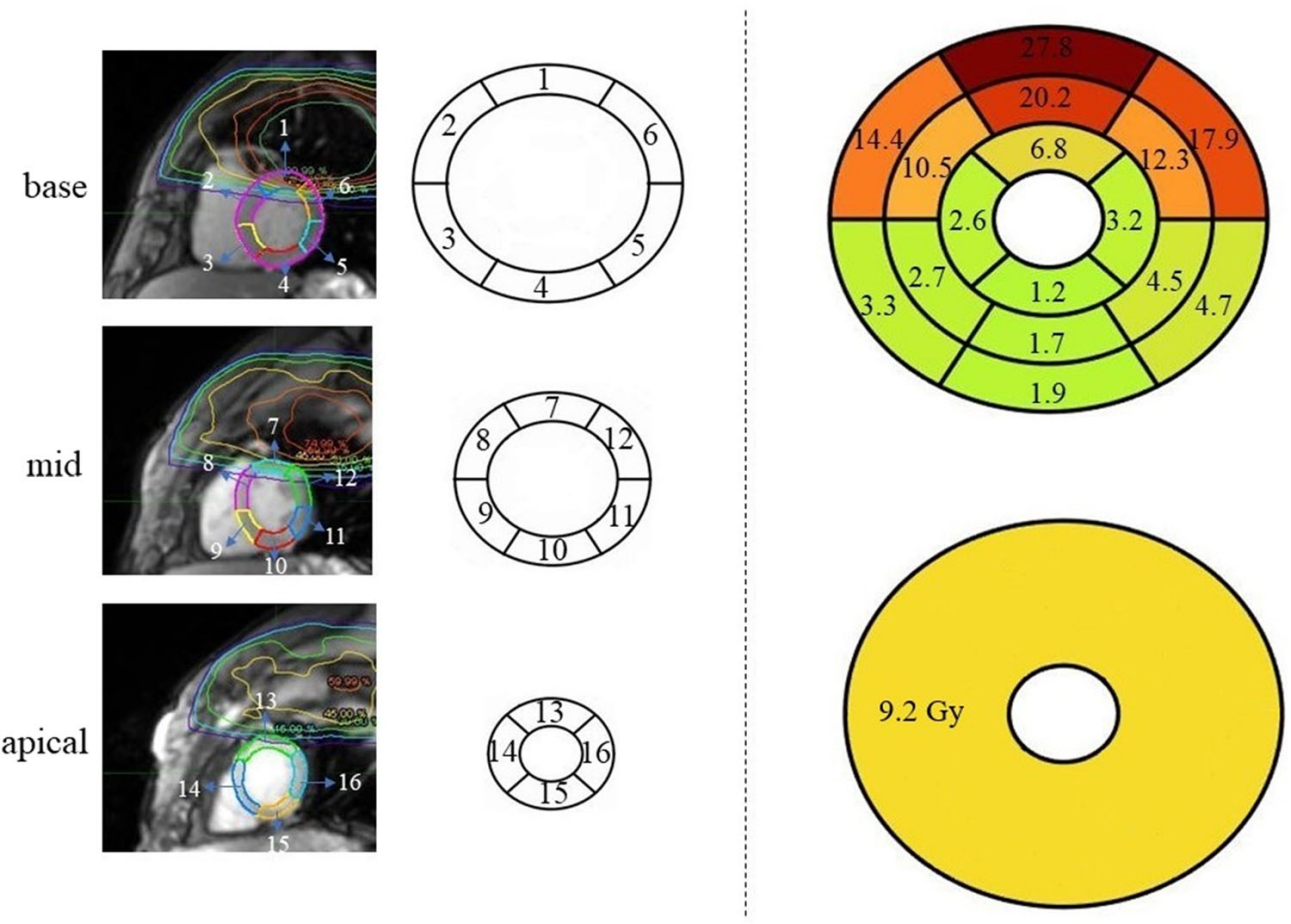
Left: a typical AHA segmentation on base, mid, apical slices of the LVM (from top to bottom). Right: top shows the heterogenous cumulative mean dose distribution on an AHA model from cardiac‐gated reference cine MR at expiration; bottom shows the mean dose measured from non‐cardiac‐gated 50% 4D‐CT (expiration). The dose values on the right‐hand‐side are averaged over the population and are in Gy

Table 4 summarizes findings from the regional analyses based on the 16‐segment AHA model and demonstrates significantly higher mean cumulative doses by cine MR at LVM regions close to the tumour (e.g., basal anterior/anterolateral/anteroseptal) compared to the mean cLV dose estimated from the non‐cardiac‐gated expiratory 4D‐CT (50%). Figure 7 illustrates these results graphically. Even though the cLV mean dose on 50% 4D‐CT was 9.2 Gy, the regional LVM dose accumulation during cardiac motion calculated mean dose values higher than 10 Gy in six segments positioned closer to the average tumour location. Additionally, the majority of the regional maximum LVM doses by cine MR were significantly less than the estimated maximum cLV dose from expiratory 4D‐CT.

**TABLE 4:** The relative maximum and mean dose comparisons between (1) cumulative cardiac‐dose and (2) 50% 4D‐CT

Sector	Corresponding anatomical position	Relative max dose (%)			Relative mean dose (%)		
		(1)	(2)	P‐value	(1)	(2)	P‐value
**AHA 1**	**basal anterior**	‐0.9	2.7	0.24	**231.5**	**11.2**	**0.0117**
**AHA 2**	**basal anteroseptal**	‐17.9	2.7	0.05	**139**	**11.2**	**0.0117**
AHA 3	basal inferoseptal	‐71.4	2.7	0.0008	‐29.2	11.2	0.2
AHA 4	basal inferior	‐82.8	2.7	0.0008	‐58.9	11.2	0.0008
AHA 5	basal inferolateral	‐59.8	2.7	0.0008	‐20.5	11.2	0.0117
**AHA 6**	**basal anterolateral**	‐20.8	2.7	0.05	**93.5**	**11.2**	**0.0046**
**AHA 7**	**mid anterior**	‐31.9	2.7	0.0011	**114.1**	**11.2**	**0.0117**
AHA 8	mid anteroseptal	‐42.8	2.7	0.0008	28.8	11.2	0.4
AHA 9	mid inferoseptal	‐81.7	2.7	0.0008	‐50.5	11.2	0.0008
AHA 10	mid inferior	‐87.7	2.7	0.0008	‐67.9	11.2	0.0008
AHA 11	mid inferolateral	‐72.4	2.7	0.0008	‐50.1	11.2	0.0008
AHA 12	mid anterolateral	‐52.6	2.7	0.0011	14.4	11.2	0.83
AHA 13	apical anterior	‐75.8	2.7	0.0008	‐33.5	11.2	0.015
AHA 14	apical septal	‐82.1	2.7	0.0008	‐65.9	11.2	0.0008
AHA 15	apical inferior	‐91.5	2.7	0.0008	‐77.9	11.2	0.0008
AHA 16	apical lateral	‐80.6	2.7	0.0008	‐63.5	11.2	0.0008

*Note*: Summary of segmental dose evaluation based on the 16‐segment AHA model between (1) the cardiac‐gated cine sequence at expiration (cumulative dose on the LVM) and (2) the non‐cardiac gated 4D‐CT at expiration (single homogeneous dose for the whole cLV). Note that AHA segments 1, 2, 6, and 7 demonstrate significantly higher relative mean doses when performing segmental analysis with cine MR compared to global analysis with clinical 4D‐CT. Relative values are measured relative to AIP dose.

## 4 DISCUSSION

Accurate consideration of cardiorespiratory motion can improve estimations of radiation dose to the heart and its substructures. In the patients in this study with tumours located primarily in the upper or middle lobes of the lung, the relative mean dose of radiation delivered to the cLV was minimum and maximum during inspiration and expiration phases, respectively. At expiration, the lowest and the highest relative mean doses on the LV or LVM occurred during systolic and diastolic phases, respectively. Even though the normalized global mean dose to the LV or LVM was not significantly different between the cumulative dose calculated from all the cardiac phases on a reference expiratory cine MR phase and the non‐cardiac‐gated 50% 4D‐CT, subregions of the LVM in proximity to the tumour received significantly higher levels of radiation than estimated by the single mean dose value derived from 50% 4D‐CT. Notably, for this pilot patient population, the global maximum dose was not significantly altered by cardiac and respiratory motion since the highest radiation iso‐dose lines crossed the LV/LVM regardless of cardiorespiratory motions in the majority of patients. Future studies in other patients with different tumour locations could present different results. The following sections provide further discussion regarding the differences in dose due to cardiopulmonary motion.

### 4.1 Respiratory motion effect on the cLV

In this study, six of eight patients had their tumour in the upper right or left lobe of the lung; the other two were in the middle lobe of the right lung. Thus, on average, the highest radiation dose was superior to the heart (i.e., closest to the anterior base of the heart). As a result, the ascending motion of the diaphragm (and heart) during expiration significantly increased the estimated mean cardiac dose relative to the inspiratory phase. In fact, the respiratory‐induced cLV displacement was noted to be maximum in the SI direction (0.4 cm) in agreement with previous studies.^8^ On the other hand, the relative maximum dose did not show significant differences between respiratory phases since at least part of the heart always remained in the high‐dose region regardless of the respiratory phase due to the proximity of the tumour to the heart. Overall, these results suggest that the delivery of radiation for tumours primarily superior to the heart should consider avoiding/minimizing delivery during expiration, when possible, in order to decrease the cardiac dose. Further studies are required to test and verify this approach.

### 4.2 Cardiac motion effect on the LVM/LV at expiration

Similar to the effect of respiratory motion, the close proximity of the tumour and basal/anterior regions of the heart led to elevated estimations of LVM dose during diastole (i.e., during maximum LV volume; cardiac phases 1–2) compared to systole (i.e., minimum LV volume; phases 8–10). That is, in this pilot population, LV contraction generally moved the anterobasal wall away from higher dose regions, but LV filling moved the wall toward the higher doses. The largest cardiac‐induced LV displacement occurring in the AP and SI directions, along with the observation of a smooth variation of maximum and mean dose during the cardiac cycle, support this interpretation (Figure 4). Consistent with these findings, previous studies have reported variations in dose to the LV/LVM during systole and diastole that depend on the patient population and tumour's location.^26^, ^27^, ^28^ Despite this difference between doses at systole and diastole, no significant dose differences were noted between the non‐cardiac gated 50% 4DCT and systole or diastole at expiration since the cLV volume on 4D‐CT remains roughly between systolic and diastolic LV volumes (due to the lack of cardiac gating). Also, relative maximum and mean LV/LVM doses over the cardiac cycle were all positive, over the population, since the cardiac cycle was monitored during expiration breath‐hold when the dose is already higher than the AIP dose.

Similar to the respiratory motion, there was no significant difference in relative maximum dose on the LVM between systole and diastole because of consistent overlap of high dose regions with at least part of the LVM regardless of the phase of the cardiac cycle. However, unlike respiratory motion, it is unlikely that radiation can be delivered quickly enough to only deliver dose during systole. Similar findings were noted for the LV except that mean dose values were lower due to its larger volume compared to the LVM (which excludes the blood pool).

### 4.3 Cardiac vs. respiratory motion effect

**FIGURE 8:**
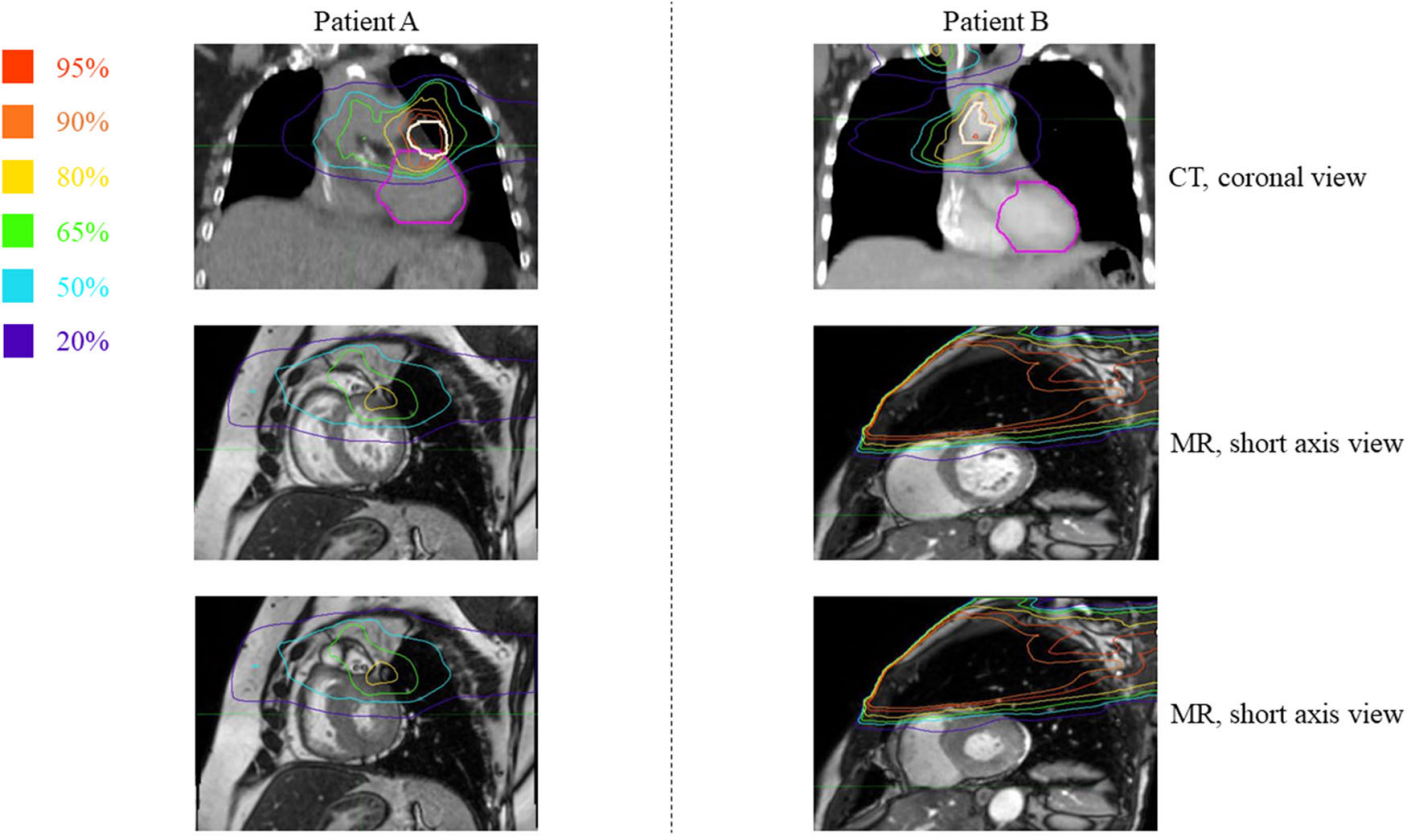
Top row: Average intensity projection (AIP) dosimetry map. Middle row: diastolic phase. Bottom row: systolic phase. For patient A, the higher isodose lines are in close proximity to the heart; they cover the LV irrespective of the cardiac cycle. For patient B, the isodose lines are far from the heart; their cardiac coverage is higher at diastole (right middle) compared to systole (right bottom). The % prescribed dose color labels are shown on the top left. Pink and white contours are LV and PTV, respectively

Similar amplitudes of cLV and LV displacement due to respiratory motion (0.6 cm) and cardiac motion (0.7 cm) (nonsignificant difference, *P* = 0.79) suggests that LV dose variations can be just as impacted by the cardiac motion as the respiratory motion. The dose distribution comparison between respiratory motion with intrinsic cardiac movement (i.e., without cardiac gating) versus cardiac motion at expiratory breath‐hold showed that the differences from lowest to highest relative mean dose through the respiratory and cardiac cycles reached approximately 30.7% and 26.9%, respectively, while the relative maximum dose differences across the cycles were 57.7% for the cardiac cycle but only 10.1% for the respiratory cycle. Even though, the relative maximum dose on cine MR was not statistically significant between systole and diastole (*P* = 0.074), a few patients with tumours located near the heart displaced the LV/LVM into high dose regions during diastole and resulted in a sharp increase of maximum dose at diastole (see Figure 8), with relative dose values > 200%–300% and an absolute dose difference of 18.6 Gy (see Figures 4 and 8). It is in these select patients that accurate accounting for cardiorespiratory motion may be most critical, emphasizing the patient‐specific nature of calculating local dose. It is also noted that patients with large tumours and/or tumours at very close proximity to the LV may not experience any relative maximum dose differences during cardiorespiratory motion since equivalent iso‐dose lines pass through the LV in all cardiac/respiratory phases (Figure 8). In addition, when comparing the LV to the LVM, the relative maximum dose did not change during the cardiac cycle since the location of the maximum was likely always on the cardiac wall (which is included in both the LV and LVM); however, the relative mean dose varied between LV and LVM by as much as 12.5% since the LVM excludes the blood pool. Since the LVM focuses solely on the cardiac muscle itself, the LVM dose may provide a more clinically relevant metric of potential damage than LV dose; however, future studies will be required to explore this hypothesis.

In terms of absolute dose, the population maximum dose variation from the lowest to highest reached 2.8 Gy (range: 0.0–6.6 Gy) and 3.1 Gy (range: 0.2–8.5 Gy) for the respiratory motion and cardiac motion, respectively, while the absolute mean dose varied between 2.7 Gy (range: 0.0–7.2 Gy) and 2.0 Gy (range: 0.2–5.6 Gy) for the respiratory and cardiac motion, respectively. Clinically, it has been shown that in lung cancer patients, the adjusted hazard ratio for major adverse cardiac events increases at a rate of 1.05/Gy of radiation to the heart.^29^ Therefore, it is critical to minimize the radiation dose to the heart and its substructures and to account for the factors (e.g., cardiopulmonary motion) that can impact the dose delivered to the heart during RT.

### 4.4 Cumulative LVM and LV dose over the whole cardiac cycle at expiration

Comparing cumulative doses over the cardiac cycle, the new method using expiratory cine MR that accounts for cardiac motion trended toward estimating greater relative maximum and mean doses to the entire LVM and LV than using the non‐cardiac gated expiratory 4D‐CT for the cLV; however, it did not reach significance (*P* > 0.17). Nevertheless, comparisons of multiple regional relative maximum and mean doses using the cine MR method were significantly different than the single maximum and mean values typically calculated and reported from 4D‐CT (Table 4). For example, the AHA sectors closest to the tumour and high dose regions (basal anterior/anteroseptal/anterolateral segments and mid anterior segments: sectors 1, 2, 6, 7) recorded relative cumulative mean dose values that were significantly greater than the mean cLV dose from non‐cardiac gated 4D‐CT (93.5%–231.5% vs. 11.2 %, *P* < 0.0117).

The regional dose analysis by cine MR provides a superior assessment of the heterogeneity of the dose distribution within the LVM that is typically unavailable by the standard non‐contrast 4D‐CT method (Figure 7). The results suggest that the 4D‐CT method significantly underestimates dose to the most exposed segments of the LVM (e.g., the anterior base in this population) and significantly overestimates dose to the least exposed regions (e.g., the apex). Clinically, this new method for quantifying local dose to individual cardiac subregions opens the door for evaluation of more accurate dose thresholds for predicting focal cardiac toxicity or for exploring the number of subregions that must be damaged before global cardiac dysfunction becomes measurably compromised. It also may improve patient‐specific risk assessments by taking into account each patient's unique and potentially regionally heterogeneous cardiac function (e.g., patient‐specific effects of radiating a region of the heart that has already experience past damage by previous infarction). Furthermore, the quantification of local radiation dose will allow for new spatial correlations to techniques for assessing local radiation‐induced cardiac toxicity (e.g., T1/T2 mapping, late gadolinium enhancement, focal strain analyses, etc.). Finally, in addition to improvements in risk‐stratifying radiotherapy for cancer treatments, enabling and quantifying motion‐resolved dosing to regional segments of the LVM is a critical need for implementing effective patient‐specific cardio‐ablative stereotactic treatments (e.g., for treating refractory arrhythmias).^9^

### 4.5 Limitations

There are a few limitations associated with this study. The dose on the cardiac scans was not recalculated; instead, it was transformed (rigidly on non‐rigidly) from the original dose calculation. However, we do not expect that the error in dose would be important enough to change any of the observations and trends made in the analyses. As previously noted, the effects of accounting for cardiorespiratory motion on local dose depend on the location of the tumour and planned radiation, both of which may be variable between patients. A larger cohort of patients with more variation in tumour location will improve the robustness and general applicability of this analytical approach and final conclusions. We also note that the breath‐hold MR images in this study were only acquired during expiration. A full evaluation of the effect of respiratory motion on estimations of cardiac dose will require the future acquisition of data at both inspiration and expiration. Finally, the extent to which the DIR is able to capture the internal shear and twist of the LVM during the cardiac cycle is unknown. Future studies are required to evaluate the uncertainty of the resulting regional dose estimates relative to the tracking of the division of the 16 segments of the AHA model through each phase of the cardiac cycle. Regarding the observed dosimetric trends, we acknowledge that the small sample size of the patient population in this study limits the power of the statistical analyses.

## 5 CONCLUSION

We have implemented a workflow to evaluate the effects of cardiac and respiratory motion on the LV and LVM delivered dose. Several trends emerged from our analyses. In lung cancer patients with tumours in the upper and middle lobes of the lung, cardiopulmonary motion can significantly affect the dose delivered to the LV/LVM (e.g., higher doses at expiration and diastole compared to lower doses at inspiration and systole). Notably, estimations of global cumulative LVM mean dose changed more than maximum dose when accounting for cardiac motion. Differences in estimated dose using the cine MR method compared to the original 50% 4D‐CT were most evident using regional analysis, with those regions closest to the target of radiotherapy demonstrating significantly greater exposure than predicted by global assessments from 50% 4D‐CT. For this study, the highest doses were predicted in the anterobasal and anteroseptal‐basal walls during diastole and expiration.

High resolution and cardiorespiratory‐gated MR methods capable of quantifying regional cardiac radiation exposure and simultaneously accounting for cardiac and respiratory motion could provide improved insight and risk stratification for future patients undergoing thoracic radiotherapy, thereby improving both clinical care and mechanistic insight into the correlation of focal radiation dose and local physiologic response.

## CONFLICT OF INTEREST

On behalf of all authors, the corresponding author states that there is no conflict of interest.

## ETHICS APPROVAL

This study was performed in line with the principles of the Declaration of Helsinki. Approval was granted by the Virginia Commonwealth University institutional review board (IRB).

## AUTHORS’ CONTRIBUTION

All authors contributed to the study conception and design. Material preparation and data analysis were performed by Alireza Omidi. The first draft of the manuscript was written by Alireza Omidi, all authors commented on previous versions of the manuscript. All authors read and approved the final manuscript. Mihaela Rosu‐Bubulac and John Wilson are co‐senior authors for this project.

## Data Availability

The raw data that support the findings of this study are available upon formal request to the corresponding author.
